# Diagnostic accuracy of a digital fundus photographic system for detection of retinopathy of prematurity requiring treatment (ROP-RT)

**DOI:** 10.1371/journal.pone.0201544

**Published:** 2018-07-31

**Authors:** Phanthipha Wongwai, Sirinya Suwannaraj, Somkiat Asawaphureekorn

**Affiliations:** Department of Ophthalmology, Faculty of Medicine, Khon Kaen University, Khon Kaen, Thailand; Universidad Miguel Hernandez de Elche, SPAIN

## Abstract

**Objectives:**

To evaluate the diagnostic accuracy of a digital fundus photographic system that consists of taking fundus photographs by a trained technician using a RetCam^®^ shuttle and interpreting fundus images by an expert to detect Retinotapthy of Prematurity requiring treatment (ROP-RT) which defined as type I ROP according to the Early Treatment for ROP study (ETROP).

**Materials and methods:**

One hundred infants were examined by (1) an expert ophthalmologist experienced in ROP care using indirect ophthalmoscopy; (2) digital wide-field imaging by a trained technician using a RetCam^®^ shuttle and images were sent remotely for interpretation by two ophthalmologists experienced in ROP care (Reader A, and Reader B); and (3) local ophthalmologists using indirect ophthalmoscopy. The diagnostic acurracy consisting of sensitivity, specificity, positive predictive value (PPV), negative predictive value (NPV), positive likelihood ratio (LR+), and negative likelihood ratio (LR-) were calculated. Agreement between all examiners and readers were evaluated.

**Results:**

A total of 100 infants (mean gestational age 31.1 weeks, mean birth weight 1,511.1 grams) participated in the study. Nine infants were classified as ROP-RT. Reader A and B had very good agreement in detection of ROP- RT (Kappa 1.00, 95% CI 1.00, 1.00). For reader A, diagnostic performance parameters (95% confidence intervals) for detecting ROP-RT were; sensitivity 100.0% (66.4, 100.0), specificity 97.8% (92.1, 99.7), PPV 81.8% (48.2, 97.7), NPV 100.0% (95.8, 100.0), LR+ 44.5 (11.3, 175.2), and LR- 0.1 (0.0, 0.8). For reader B these were; sensitivity 100.0% (66.4, 100.0), specificity 95.6% (89.0, 98.8), PPV 69.2% (38.6, 90.9), NPV 100.0% (95.8, 100.0), LR+ 22.5 (8.6, 58.6), LR- 0.1 (0.0, 0.8). No adverse events were reported.

**Conclusions:**

Diagnosis of ROP-RT from RetCam^®^ images taken by trained technicians and evaluated remotely by an expert ophthalmologist had good diagnostic accuracy for screening purposes.

## Introduction

Retinopathy of prematurity (ROP) is one of the leading causes of blindness in prematurely born children. Prompt diagnosis and treatment is key in the management of this potentially blinding disease. Carefully timed retinal examinations should be done by an ophthalmologist with experience in the examination of tiny babies, as described in the international recommendations [1]. In practice, not all countries and regions can provide enough ophthalmologists qualified as previously described especially in the developing countries. For these reasons, not all cases with ROP requiring treatment (ROP-RT) are picked up in due time, and even if they are, some babies have to be referred to a center to be examined by an ophthalmologist who expert in ROP care. Often these fragile infants still have an endotracheal tube and many catheters, so that transportation may put them at an additional risk. Another important issue is the cost of transportation across the city or country that may be required repeated times until the retinas are mature.

There are many articles that mentioned the use of wide field digital fundus cameras that could capture the fundus images and send to expert ophthalmologists for making the diagnosis [2–22]. These infants therefore do not have to travel to meet the expert, but there is still some debate about the role of this method for screening purposes.

The objective of this study was to evaluate the diagnostic performance of digital fundus photographic system for detection of ROP requiring treatment (ROP-RT, according to the ETROP study [23]), ROP of any stage (ROP-AS) and the presence of plus disease (Plus) when a trained technician take the photos and an expert ophthalmologist make the diagnosis. In addition, the diagnostic performance of fundus examination with indirect ophthalmoscopy by the local ophthalmologists who were not the expert in ROP care were also evaluated. In provincial hospitals, the local ophthalmologists are those who usually perform the ROP screening.

## Materials and methods

This study was approved by the Khon Kaen University Ethics Committee in Human Research before beginning of the research procedures.

### Study population

The participants were: (1) preterm infants who were referred from other hospitals to Srinagarind Hospital, Khon Kaen for ROP diagnosis and treatment, (2) infants for whom ROP screening was requested according to their own criteria from 4 provincial hospitals in the northeast of Thailand including Khon Kaen Hospital, Kalasin Hospital, Roi-et Hospital, and Mahasarakham Hospital. The exclusion criteria were: (1) an infant with an unstable condition as determined by the pediatrician, (2) an infant with a history of hyperglycemia, (3) an infant with ocular infection.

### Study procedure

After written informed consent was obtained from the parents, the infant’s pupil was dilated with 1% Mydriacyl eye drops every 5 minutes for 3 times alternated with 2.5% phenynephrine eye drops every 5 minutes for 3 times. The examination started 30 minutes after the last eye drop. The infant was swaddled, fed with 24% sucrose solution through a pacifier for reducing pain and stress. The examination was started by putting in the anesthetic eye drop, inserting lid speculum and performing the retinal examination using indirect ophthalmoscopy with 28 diopters lens and scleral indentation by the expert ophthalmologist who had experience in ROP care. This is supposed to be the practical reference standard. The infant was then allowed to rest for 10 minutes. The second examination was the fundus photography by using a RetCam^®^ shuttle (Clarity Medical Systems, Inc., Pleasanton, CA). Four images were captured for each eye including; the anterior segment, posterior pole, nasal and temporal retina. The fundus images were sent to two ophthalmologists who were experienced in ROP care (reader A and B) for making the diagnosis via a CD without other identifiable information. The infant was then allowed to rest for another 10 minutes. The last examination was indirect ophthalmoscopy with 20 or 28 diopters lens and scleral indentation by the local ophthalmologists of each hospital who were general ophthalmologists and did not have much experience in ROP care. For infants who were referred to Srinagarind Hospital, all the above procedures were done, but the diagnosis of the local ophthalmologists were identified from the referral document. The duration from the examination at the referral hospitals until the examination at Srinagarind Hospital should not be more than 48 hours. Each ophthalmologist and the photographer were masked to the diagnosis and the identifiable information of the infants. ROP-RT was classified according to the ETROP study as type I ROP (zone I, any stage, with plus disease; zone I, stage 3 with or without plus disease; or zone II, stage 2 or 3, with plus disease) [23].

### Statistical analysis

The unit of analysis for ROP-RT was at the level of individuals; an infant would be classified as ROP-RT if one or both eyes were classified as ROP-RT. For ROP-AS and Plus, to avoid for correlation between eyes, one eye was randomly selected from each infant for statistical analysis.

All data were analyzed using R software version 3.4.1 [24]. Demographic data were described as mean, standard deviation (SD), median, minimum and maximum values. Diagnostic performance parameters were calculated using epiR package in R [25]. The following diagnostic performance parameters and their 95% confidence intervals (95% CI) were calculated; sensitivity, specificity, positive predictive value (PPV), negative predictive value (NPV), positive likelihood ratio (LR+), and negative likelihood ratio (LR-). The 95% CI for sensitivity, specificity, PPV, and NPV were calculated with the exact confidence intervals method [26]. Confidence intervals for LR+ and LR- were based on formulas provided by Simel et al [27]. Since there were cells with zero value in two tables, 0.05 was added to all cells in those tables to solve the mathematical calculation of LR-. This method had been proposed by Haldane [28] and Anscombe [29].

Likelihood ratio (LR) was included in the analysis because it was a powerful tool for clinical decision making [30]. LR is the ratio of the probability of a given test result in patients with the disease to the probability of the same test result in patients without the disease. It incorporates both sensitivity and specificity into a single measure. For LR+, it is sensitivity/(1 –specificity). For LR-, it is (1 –sensitivity)/specificity.

LR is stable to changes in prevalence of disease as opposed to PPV and NPV. PPV and NPV calculated in a setting with a certain prevalence of the outcome cannot be applied to other settings with different prevalence. Therefore LR+ and LR- are recommended to use for calculation of PPV and NPV respectively based on the specific prevalence or pretest probability. This can be easily done with a standard Fagan’s nonogram [31] or a modified one [32]. LR for a given test result indicates how much that result will raise (if LR > 1) or lower (if LR < 1) the pretest probability. If LR = 1, the test is of no utility. For a rough guide, LR of > 10 or < 0.1 generate large and often conclusive changes from pretest to posttest probability [33].

The level of agreement between each method of examination was reported as Cohen’s kappa using psych package in R [34]. Confidence intervals for kappa were calculated using methods suggested by Fleiss [35].

## Results

**Fig 1:**
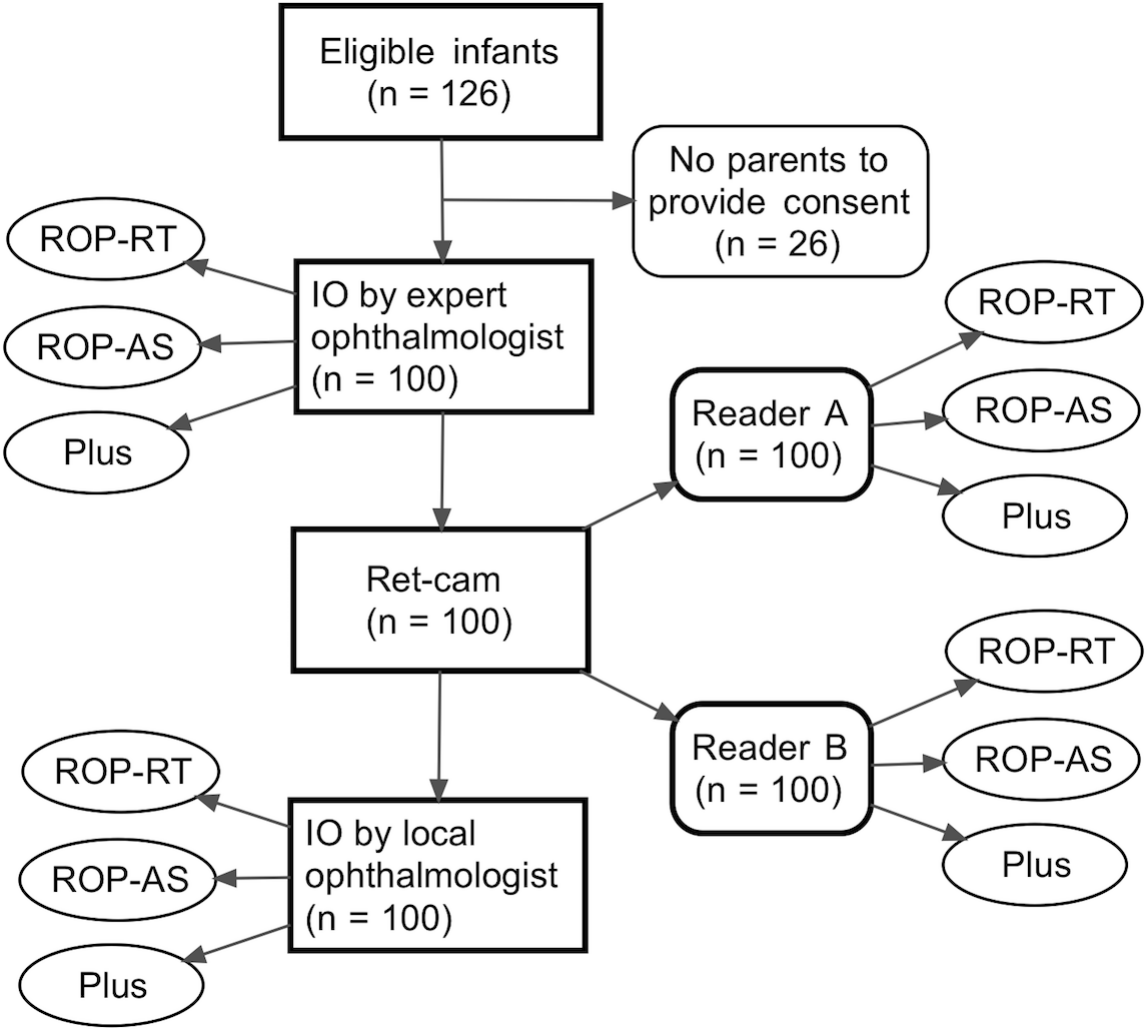
Study flow diagram. Ret-cam = trained technician took fundus photograph and sent to expert ophthalmologist (reader A and reader B) for interpretation; IO by expert ophthalmologist = Indirect ophthalmoscopy by expert ophthalmologist (reference standard); IO by local ophthalmologist = Indirect ophthalmoscopy by local ophthalmologist; ROP-RT = ROP requiring treatment; ROP-AS = ROP of any stage; Plus = presence of plus disease.

From May to September 2013, 126 infants met the eligible criteria. The parents of 26 infants were not available to provide their informed consent. A study flow diagram was shown in Fig 1.

The characteristics of all enrolled 100 infants and that were diagnosed as ROP were shown in Table 1. There were 18 infants who were referred to Srinagarind Hospital while 82 were infants from the 4 provincial hospitals. According to the expert ophthalmologist (reference standard), 9 of 100 infants were diagnosed as ROP-RT (2 infants were those referred to Srinagarind Hospital, and 7 infants were those from the 4 provincial hospitals). In addition, 31 infants were diagnosed as ROP-AS, and 8 infants as Plus. For ROP-AS, 9 infants (29%) were diagnosed as stage 1 ROP, 20 infants (65%) as stage 2 ROP, 1 infant (3%) as stage 3 ROP, and 1 infant (3%) as stage 4 ROP. There was no stage 5 ROP.

**Table 1:** Baseline data of all enrolled infants and infants with ROP.

	Mean	SD	Min	Median	Max
**All enrolled infants (n = 100)**					
Gestational Age (weeks)	31.1	3.0	24.0	32.0	40.0
Birth weight (grams)	1,511.1	421.2	760.0	1,565.0	2,930.0
Age at examination (weeks)	7.0	4.8	3.0	4.0	32.0
**Infants with any stages of ROP (n = 31)**					
Gestational Age (weeks)	29.8	2.7	24.0	31.0	34.0
Birth weight (grams)	1,330.9	358.1	760.0	1,320.0	2,360.0
Age at examination (weeks)	6.9	3.1	3.0	8.0	32.0

SD = standard deviation; Min = minimum; Max = maximum

Based on the diagnosis of the expert ophthalmologist as a reference standard, the number of infants in each method of examination were shown in Table 2. For ROP-RT, poor image quality precluded the interpretation in 2 infants for reader A and 1 infants for reader B. For ROP-AS, poor image quality precluded the interpretation in 2 infants for reader A. For Plus disease, poor image quality precluded the interpretation in 2 infants for reader A and 1 infant for reader B. Two infants had media opacity that precluded the evaluation of Plus disease by the local ophthalmologist.

**Table 2:** Cross tabulation of the number of infants for 3 methods of examination (Reader A, Reader B, and local ophthalmologist) against indirect ophthalmoscopy by the expert ophthalmologist (the reference standard).

Methods of examination	Test +ve		Test -ve		Total
	Expert +ve	Expert -ve	Expert +ve	Expert -ve	
**ROP-RT (9 infants)**					
Reader A	9	2	0	87	98
Reader B	9	4	0	86	99
Local oph	8	6	1	85	100
**ROP-AS (31 infants)**					
Reader A	12	1	19	66	98
Reader B	13	2	18	67	100
Local oph	24	3	17	66	100
**Plus (8 infants)**					
Reader A	4	1	4	89	98
Reader B	6	8	2	83	99
Local oph	5	3	3	87	98

ROP-RT = ROP requiring treatment; ROP-AS = ROP of any stage; Plus = plus disease; Test +ve = test positive; Test -ve = test negative; Expert +ve = positive indirect ophthalmoscopy by the expert ophthalmologist; Expert -ve = negative indirect ophthalmoscopy by the expert ophthalmologist; Local oph = indirect ophthalmoscopy by the local ophthalmologist.

The diagnostic performance of the 3 methods of examination for the 3 categories of classification including sensitivity, specificity, PPV, NPV, LR+, and LR- were calculated from the numbers in Table 2 and were shown in Table 3.

**Table 3:** Diagnostic Performance of each method of eye examination classified in 3 categories.

Methods of	Sens %	Spec %	PPV %	NPV %	LR+	LR-
examination	(95% CI)	(95% CI)	(95% CI)	(95% CI)	(95% CI)	(95% CI)
**ROP-RT**						
Reader A	100.0	97.8	81.8	100.0	44.5	0.05*
	(66.4, 100.0)	(92.1, 99.7)	(48.2, 97.7)	(95.8, 100.0)	(11.3, 175.2)	(0.00, 0.77)
Reader B	100.0	95.6	69.2	100.0	22.5	0.05*
	(66.4, 100.0)	(89.0, 98.8)	(38.6, 90.9)	(95.8, 100.0)	(8.6, 58.6)	(0.00, 0.78)
Local oph	88.9	93.4	57.1	98.8	13.5	0.1
	(51.8, 99.7)	(86.2, 97.5)	(28.9, 82.3)	(93.7, 100.0)	(6.0, 30.2)	(0.0, 0.8)
**ROP-AS**						
Reader A	38.7	98.5	92.3	77.6	25.9	0.6
	(21.8, 57.8)	(92.0, 100.0)	(64.0, 99.8)	(67.3, 86.0)	(3.5, 190.7)	(0.5, 0.8)
Reader B	41.9	97.1	86.7	78.8	14.5	0.6
	(24.5, 60.9)	(89.9, 99.6)	(59.5, 98.3)	(68.6, 86.9)	(3.5, 60.3)	(0.4, 0.8)
Local oph	77.4	95.6	88.9	90.4	17.8	0.2
	(58.9, 90.4)	(87.8, 99.1)	(70.8, 97.6)	(81.2, 96.1)	(5.8, 54.7)	(0.1, 0.5)
**Plus**						
Reader A	50.0	98.9	80.0	95.7	45.0	0.5
	(15.7, 84.3)	(94.0, 100.0)	(28.4, 99.5)	(89.4, 98.8)	(5.7, 356.1)	(0.3, 1.0)
Reader B	75.0	91.2	42.9	97.6	8.5	0.3
	(34.9, 96.8)	(83.4, 96.1)	(17.7, 71.1)	(91.8, 99.7)	(3.9, 18.5)	(0.1, 0.9)
Local oph	62.5	96.7	62.5	96.7	18.8	0.4
	(24.5, 91.5)	(90.6, 99.3)	(24.5, 91.5)	(90.6, 99.3)	(5.5, 64.5)	(0.2, 0.9)

ROP-RT = ROP requiring treatment; ROP-AS = ROP of any stage; Plus = plus disease; Sens = sensitivity; Spec = specificity; PPV = positive predictive value; NPV = negative predictive value; LR+ = positive likelihood ratio; LR- = negative likelihood ratio; 95% CI = 95% confidence interval

* To calculate LR- when there was 0 in a cell, 0.5 was added to all cells.

The agreement of classifications among reader A, reader B, local ophthalmologist, and expert ophthalmologist for ROP-RT were shown in Table 4.

**Table 4:** Agreement (Cohen’s Kappa) between each pair of examination method based on ROP-RT.

	Reader B	Local Oph	Expert Oph
	Kappa (95% CI)	Kappa (95% CI)	Kappa (95% CI)
Reader A	1.00 (1.00, 1.00)	0.75 (0.55, 0.96)	0.89 (0.74, 1.00)
Reader B	–	0.72 (0.50, 0.93)	0.80 (0.60, 0.99)
Local Oph	–	–	0.66 (0.43, 0.89)

ROP-RT = ROP requiring treatment; Expert oph = Indirect ophthalmoscopy by expert ophthalmologist (reference standard); Local oph = Indirect ophthalmoscopy by local ophthalmologist; 95% CI = 95% confidence interval

All infants successfully went through all 3 methods of examinations without any complications.

## Discussion

There were many reports from all regions around the world that mentioned about the diagnostic accuracy of digital fundus photography for the diagnosis of ROP-RT or high risk ROP [2–18,20–22,36]. This study aimed to evaluate the accuracy of this technique with the addition of examination by the local ophthalmologists who were usually performed the ROP screening in the provincial hospitals. If the accuracy of ROP examination by digital fundus photography is accurate and clinically acceptable, it will be a useful alternative ROP screening method in the area where there are work overload and insufficient expert ophthalmologists experienced in ROP care.

The infants in this study had older gestational age and higher birth weight which were different from previous studies [2–4,6,7,9–14,16,17,19–22,37–44]. This might reflect the neonatal care system in this region and might suggest the need for ROP screening in the older gestational age and higher birth weight infants.

Regarding the diagnostic accuracy of digital fundus photographic system when ROPT-RT was the target outcome, reader A and B performed better than the local ophthalmologist which was reflected by very high values of sensitivity, specificity, PPV, and NPV (Table 3). This indicates that digital fundus photographic system is a good and accurate method for ROP screening. The local ophthalmologist missed 1 of 9 cases of ROP-RT while reader A and B did not miss any case (Table 2). This could probably be explained by the inexperience of the local ophthalmologist in examination of very small infants.

This study had similar results to the previous studies that supported the benefit of digital fundus photographic system to detect clinically significant ROP or ROP-RT [2,3,6,9,11–13,15–17,19–22,36,38–40,42,44] but our results had higher sensitivity, specificity, and LR+. The difference in instrumentation and images quality may contribute to these differences.

When the target outcome was ROP-AS, the sensitivity values of digital fundus photographic system were decreased for reader A (38.7%) and reader B (41.9%). However, the specificity values were still very good for reader A (98.5%) and reader B (97.1%) (Table 3). One explanation could be the limitation of digital fundus camera that could not capture the fundus images beyond anterior zone II and preclude the diagnosis of ROP in zone III, while the local ophthalmologist could use scleral indentation technique to visualize more peripheral retinal area. This would not be a problem because, according to the ETROP study, type 2 ROP including ROP in zone III was low risk ROP and did not require any treatment. Another possible explanation was difficulty of the readers to differentiate three dimensions nature of ridges from lines in some images.

When plus disease was the target outcome, both readers and local ophthalmologist had moderate sensitivity but high specificity with varying values (Table 3). The diagnosis of plus disease was subjective in nature even among the expert ophthalmologists, which might be the reason for variation of the diagnostic performance in our study and also previous published studies [45–47].

All the LR+ of any methods of examination (images reading remotely by reader A and B, and indirect ophthalmoscopy by the local ophthalmologist) according to any categories (ROP-RT, ROP-AS and Plus) were very high (Table 3). That is, almost all of them had the LR+ of greater than 10 (only reader B for Plus disease had LR+ of 8.5). In other words, the true positive results given by any of these methods of examination were about more than ten times the false positive. Hence, these methods were very useful in clinical decision making. For ROP-RT, the LR+ for reader A and B were much higher than the local ophthalmologist, which highlighted the usefulness of the digital fundus photographic system.

There was very good agreement among reader A, reader B and expert ophthalmologist in detection of ROP- RT from interpreting the fundus photographs compared with other methods (Table 4). This was anticipated because all of them were experts in ROP care. The agreement between local and expert ophthalmologist (kappa 0.66) was less than the agreement between reader A and expert ophthalmologist (kappa 0.89) and between reader B and expert ophthalmologist (kappa 0.80) (Table 4).

Many articles also reported similar results with this study that no difference in overall accuracy between ophthalmoscopy and telemedicine for detection of clinically significant ROP. But our study had added another aspect regarding the accuracy of currently ROP screening practice done by local ophthalmologists. Our results showed that digital fundus photographic system had better diagnostic accuracy than the local ophthalmologist for ROP-RT. However, the diagnostic performance of this method greatly affected by the quality of the fundus images. The diameter of pupils was an important factor that influenced the quality of the fundus images. There were 2 infants with poor quality images for reader A and 1 infant for reader B that precluded the categorization of ROP-RT. The size of the pupils were about 2 and 4 millimeters in these cases. We recommended that the pupil size for obtaining an interpretable image should be more than 5 millimeters. Another factor that might affect the quality of fundus images was the training of fundus imaging. Standardization of training and protocols for the photographers is the key to get a good diagnostic performance of the digital fundus photographic system. Morrison et al used 5 fundus images for telemedicine in ROP [48] while Quinn et al used 6 images [22]. Both reports found higher sensitivity to detect clinically significant ROP. Our study submitted only 4 images to the readers and still had high sensitivity and specificity in detection of ROP-RT. This could be explained by the difference in the experience of the readers. In our study, the readers were expert ophthalmologists in ROP care while Morrison and Quinn used non-physician as readers.

One of the limitation in our study was that all hospitals did not use the same standard criteria for ROP screening. The Royal College of Ophthalmologists of Thailand had developed a guideline for ROP screening but each hospital adapted the guideline according to their situation of neonatal care. Our study allowed each hospital to use their own ROP screening guideline because they might have different level of neonatal care.

Another limitation in our study was that the order of examination might affect the outcome of the last examiner (indirect ophthalmoscopy by the local ophthalmologist). The sequence of our study started with fundus examination by the expert ophthalmologist, followed by RetCam^®^ imaging, and fundus examination by the local ophthalmologist. The long process of examination might alter the clarity of the cornea causing difficulty to visualize the fundus by the local ophthalmologist.

In conclusion, the diagnosis of ROP-RT from RetCam^®^ images taken by trained technicians and evaluated remotely by an expert ophthalmologist had good diagnostic accuracy for screening purposes.

The authors listed in this study do not have any financial conflict of interest in any items mentioned in this study.
